# Analysis of the Dissolution Behavior of Theophylline and Its Cocrystal Using ATR-FTIR Spectroscopic Imaging

**DOI:** 10.1021/acs.molpharmaceut.4c00002

**Published:** 2024-05-28

**Authors:** Yuna Tatsumi, Yusuke Shimoyama, Sergei G. Kazarian

**Affiliations:** † Department of Chemical Science and Engineering, Tokyo Institute of Technology, S1-33 2-12-1 Ookayama, Meguro-ku, Tokyo 1528550, Japan; ‡ Department of Chemical Engineering, Imperial College London, London SW7 2AZ, United Kingdom

**Keywords:** ATR-FTIR spectroscopic imaging, crystal engineering, cocrystal, theophylline, dissolution mechanism

## Abstract

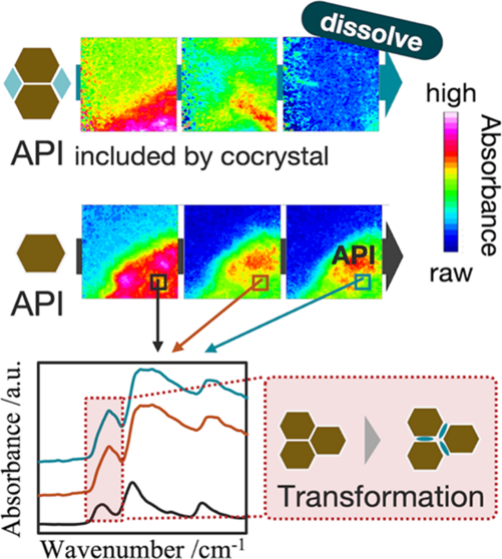

Attenuated total reflection-Fourier transform infrared (ATR-FTIR) spectroscopic imaging is a powerful tool to visualize the distribution of components, and it has been used to analyze drug release from tablets. In this work, ATR-FTIR spectroscopic imaging was applied for observing the dissolution of molecular crystals from tablet compacts. The IR spectra provided chemically specific information about the transformation of crystal structures during the dissolution experiments. Theophylline (TPL) anhydrate and its cocrystals were used as model systems of molecular crystals. The IR spectra during the dissolution of TPL revealed information about the crystal structure of TPL, which transformed from anhydrate to monohydrate in water. During a dissolution test of a model cocrystal system, it was suggested that an active pharmaceutical ingredient (API) and a coformer were dissolved in water simultaneously. The IR spectra that were acquired during the dissolution of a cocrystal tablet showed new spectral bands attributed to the API after 5 min. This suggested that the precipitation of API was observed during the dissolution experiment. Measurements from ATR-FTIR spectroscopic imaging can visualize the drug release from the tablet and determine the transformation of molecular crystals during their dissolution. These results will have an impact on clarifying the dissolution mechanism of molecular crystals.

## Introduction

More than 80% of the pharmaceutical products on the market are solid dosage forms for oral administration, which are typically sold as tablets and capsules.^1^ For effective solid dosage forms, it is necessary to demonstrate the appropriate solubility and bioavailability of active pharmaceutical ingredients (APIs). Furthermore, considerations such as reduction of operation cost in manufacturing, product safety, and product stability during storage are essential for marketed products. In particular, APIs in solid dosage forms need to have appropriate solubility in aqueous environments. However, drug development faces an issue, namely, most of the APIs show low solubility in water.^2^

Crystal engineering approaches have been focused on improving the solubility of the API.^3^ Typical examples of crystal engineering techniques include amorphous, salt, and cocrystal.^4−6^ Amorphous is a disordered solid that lacks a regular crystalline structure. The amorphous form gives a higher solubility and a faster dissolution rate in aqueous media than the crystalline state. Amorphous APIs are not thermodynamically stable, and they can undergo crystallization to their more stable forms. Amorphous solid dispersion, which is a formulation of amorphous APIs and a carrier material such as a polymer, is often used in order to maintain the physical stability of amorphous APIs.^7^ A salt is a composite crystal composed of an API and another molecule by ionizing an acid or base functional group. Salt may dissociate and convert to the nonionized form of APIs due to storage conditions such as humidity, which occurs because salt, including the nonionized form, shows a lower solubility in water than pure salt. It is necessary to store pharmaceutical salt under suitable conditions and maintain the stability of the salt.^8^ Moreover, a cocrystal is a composite crystal composed of API and another molecule called a coformer through the covalent bond. APIs without a dissociable group can be applied to cocrystals, which is one of the main reasons why cocrystals have been focused on particularly. During the dissolution of a cocrystal, the intermolecular bond of the cocrystal can be dissociated immediately in a medium and the APIs can become the metastable form of APIs. The metastable form of API shows a higher solubility than the original form of API. In order to keep the high solubility of APIs, it is required to maintain the metastable form of APIs for long periods of time.^9^ Hence, the dissolution profile of an API in a cocrystal depends on the transformation of the API’s crystal structure during the dissolution. To achieve the appropriate dissolution profile of the cocrystal forms, it is necessary to provide the formulation design based on the transformation mechanism of API’s crystal structure during dissolution.

The dissolution behavior of an API can be characterized by UV–visible spectroscopy (UV–vis) and high-performance liquid chromatography (HPLC). Both methods are commonly used to measure the solubility of the API in water. The solubility measurements by UV–vis or HPLC indicate that the solubility of the API is improved by crystal engineering techniques such as amorphous,^10^ salt,^11^ and cocrystal.^11,12^ The solubility measurements by HPLC, provided that the salt is composed of olanzapine and 3-hydroxy benzoic acid, showed approximately twice the solubility of pure olanzapine in the first 2 h.^11^ Therefore, solubility measurements can be useful to understand the dissolution property of APIs. However, solubility measurements provide little knowledge to clarify the transformation mechanism of the crystal structure during the dissolution process.

Attenuated total reflection-Fourier transform infrared (ATR-FTIR) spectroscopic imaging has been used to analyze the drug release from pharmaceutical products.^13−21^ It provides the FTIR spectra of every point in a measurement area and visualizes the distribution of components.^22^ It is a nondestructive and in situ chemical imaging method. These characteristics of ATR-FTIR spectroscopic imaging are effective for analyzing drug release from pharmaceutical products. In particular, ATR-FTIR spectroscopic imaging has been applied to the dissolution tests of APIs in amorphous solid dispersions.^13,14,19,23−26^ In amorphous solid dispersions, the time dependence of ATR-FTIR images suggested that the dissolution rate of API was controlled by both the type^17,23^ and weight^19,25^ of the matrix. For example, soluplus and poly(vinylpyrrolidone) (PVP) were applied to the matrix materials of amorphous solid dispersions including aprepitant as an API.^17^ The time dependences of ATR-FTIR images have shown that the recrystallization of aprepitant was observed only in the amorphous solid dispersion, including PVP as the matrix.^17^ It can be proposed that a weak interaction between aprepitant and PVP results in a rapid dissolution of PVP, and thus, recrystallization of the aprepitant occurs. It means that an amorphous solid dispersion needs to select an appropriate carrier material for each API. ATR-FTIR spectroscopy has been applied to understand the drug–carrier interactions on drug release.^26^ Therefore, the images from ATR-FTIR spectroscopic imaging can be useful to analyze the crystal structure transformation during the dissolution of APIs from an amorphous solid dispersion.

In this work, we applied ATR-FTIR spectroscopic imaging to the dissolution analysis of pharmaceutical molecular crystals. Real-time monitoring with ATR-FTIR spectroscopic imaging can visualize the dissolution behavior of APIs on the tablet. The raw IR spectra data given by the ATR-FTIR spectroscopic imaging measurement can be used for peak shift analysis. It can be used to analyze chemically specific information about the transformation of a crystal structure during dissolution. The measurement data from ATR-FTIR spectroscopic imaging are expected to visualize the drug release and analyze the dissolution process based on the transformation of the crystal structure. To analyze the dissolution process of pharmaceutical molecular crystals, dissolution tests were carried out in two types of solid forms: the molecular crystal of a single component and that composed of multiple components. We used theophylline (TPL) anhydrate as an API in the dissolution test for the molecular crystal of a single component. It is known that the crystal structure of TPL transformed from anhydrate to monohydrate in water.^27−29^ The dissolution test of TPL anhydrate can become a model case to detect the transformation of a molecular crystal during dissolution. Furthermore, we used a cocrystal in the dissolution test for the molecular crystal composed of multiple components. In this work, TPL and nicotinamide (NA) were selected as the API and the coformer, respectively. This work is expected to expand the scope for applications of ATR-FTIR spectroscopic imaging because it has already been applied to analyze the formation process of cocrystals,^30,31^ but it has not been applied to analyze the dissolution behavior of cocrystals to our knowledge.

## Experimental Section

### Materials

TPL (purity over 99.0%), NA (purity over 98.0%), and ethanol (purity over 99.5%) were supplied by Fuji Film Wako Pure Chemical Industries Ltd.

### Preparation and Characterization of the Cocrystal

The cocrystal was fabricated by liquid-assisted grinding. TPL (1.0 mmol) as the API and NA (1.0 mmol) as the coformer were added to the vessel containing ethanol (0.3 mL). The mixture was stirred at 300 rpm for 2 h. After being stirred, the remaining powder was analyzed by powder X-ray diffraction (PXRD) (Rigaku Corp.) and Fourier transform infrared spectrometry (FTIR) (JASCO Co., Ltd.) to characterize the crystal structure and confirm cocrystallization. The PXRD measurement was conducted with a step size of 0.02° and a scanning speed of 2.0° min^–1^.

### Tablet Dissolution Test with ATR-FTIR Spectroscopic Imaging

**Figure 1:**
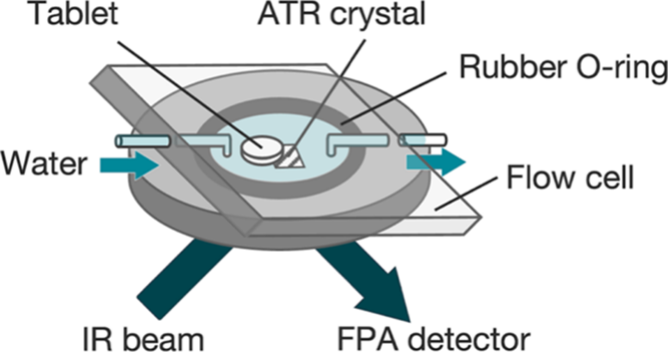
Schematic diagram for dissolution experiments using ATR-FTIR spectroscopic imaging.

Figure 1 shows the experimental apparatus, including a flow cell designed by a previous study^16^ and an ATR-FTIR spectroscopic imaging system. The ATR-FTIR spectroscopic imaging system was composed of an FTIR spectrometer (Bruker UK), a focal plane array (FPA) detector with 64 × 64 pixels (Santa Barbara Focalplane), and Golden Gate ATR accessory (Specac Ltd.). Golden Gate ATR accessory features a diamond ATR crystal as an internal reflection element (IRE) material. It has an imaging area of 0.6 × 0.55 mm^2^ with a spatial resolution of 15 μm. The tablet with 3 mm diameter, which was fabricated according to a previous study,^32^ was placed on the diamond ATR crystal surrounded by a rubber O-ring. A flow cell was placed on the ATR crystal. The flow cell was sealed by the rubber O-ring and four-point screws, which can allow sufficient contact between the tablet and IRE. To make water flow into the flow cell, two rubber tubes were connected to the inlet and outlet sides of the flow cell separately. A syringe was connected to the rubber tube on the inlet side. The syringe allowed water to flow into the flow cell from a pump. The flow rate of water was set to 0.5 mL min^–1^. ATR-FTIR spectroscopic imaging measurements were performed at 4 cm^–1^ resolution using 32 scans in the spectral range of 3900–900 cm^–1^. The acquisition time was about 160 s. Dry measurement was defined as the time before water flowed into the flow cell. The dissolution test was started when water flowed into the flow cell, which was defined as 0 min. ATR-FTIR images of TPL and NA were obtained by integrating the absorbance of specific bands (TPL: C=O stretch (1736–1688 cm^–1^)^33^ and NA: C–C stretch (1447–1370 cm^–1^)^34^ across all pixels using OPUS software (Bruker UK).

### Quantitative Analysis Based on ATR-FTIR Spectroscopic Imaging

Quantitative analysis for tablet dissolution based on ATR-FTIR spectroscopic images was processed by the following three steps. First, the integrated absorbance values of each pixel were calculated for the ATR-FTIR spectroscopic image. TPL and NA were applied to the peak in the specific bands (TPL: C=O stretch (1736–1688 cm^–1^)^33^ and NA: C–C stretch (1447–1370 cm^–1^).^34^ Second, the integrated absorbance values were averaged for each image individually. Finally, the normalized integrated absorbance was calculated as follows:
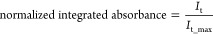
1where *I*_t_ is the average value of integrated absorbance and *I*_t_max_ is the maximum value for the average of integrated absorbance.

## Results and Discussion

### Dissolution Test for the Molecular Crystal of a Single Component

**Figure 2:**
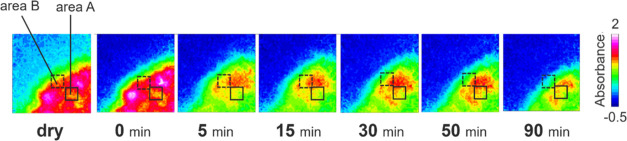
ATR-FTIR spectroscopic images of the distribution of theophylline during dissolution.

**Figure 3:**
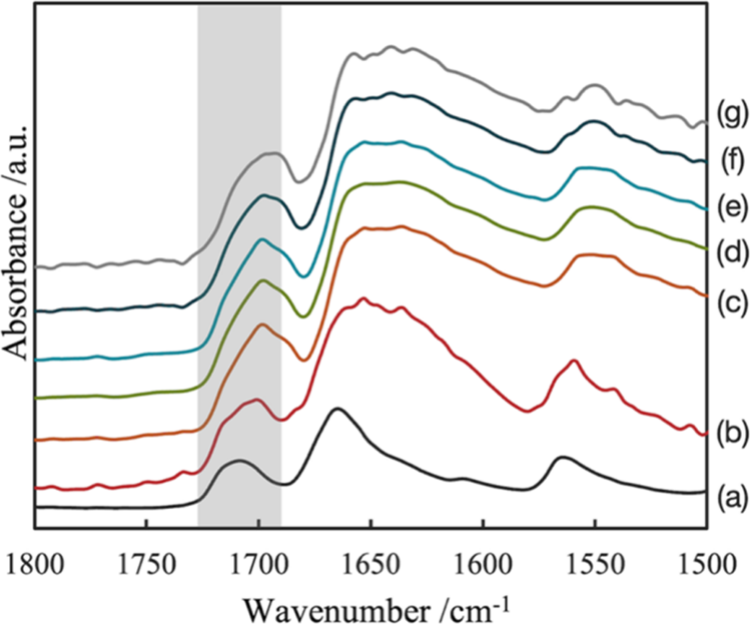
Time dependence of IR spectra. IR spectra at each time condition were the average of all spectra extracted within area *A* in Figure 2. The peak around 1736 and 1688 cm^–1^ is guided by the gray bar: (a) dry, (b) 0 min, (c) 5 min, (d) 15 min, (e) 30 min, (f) 50 min, and (g) 90 min.

**Figure 4:**
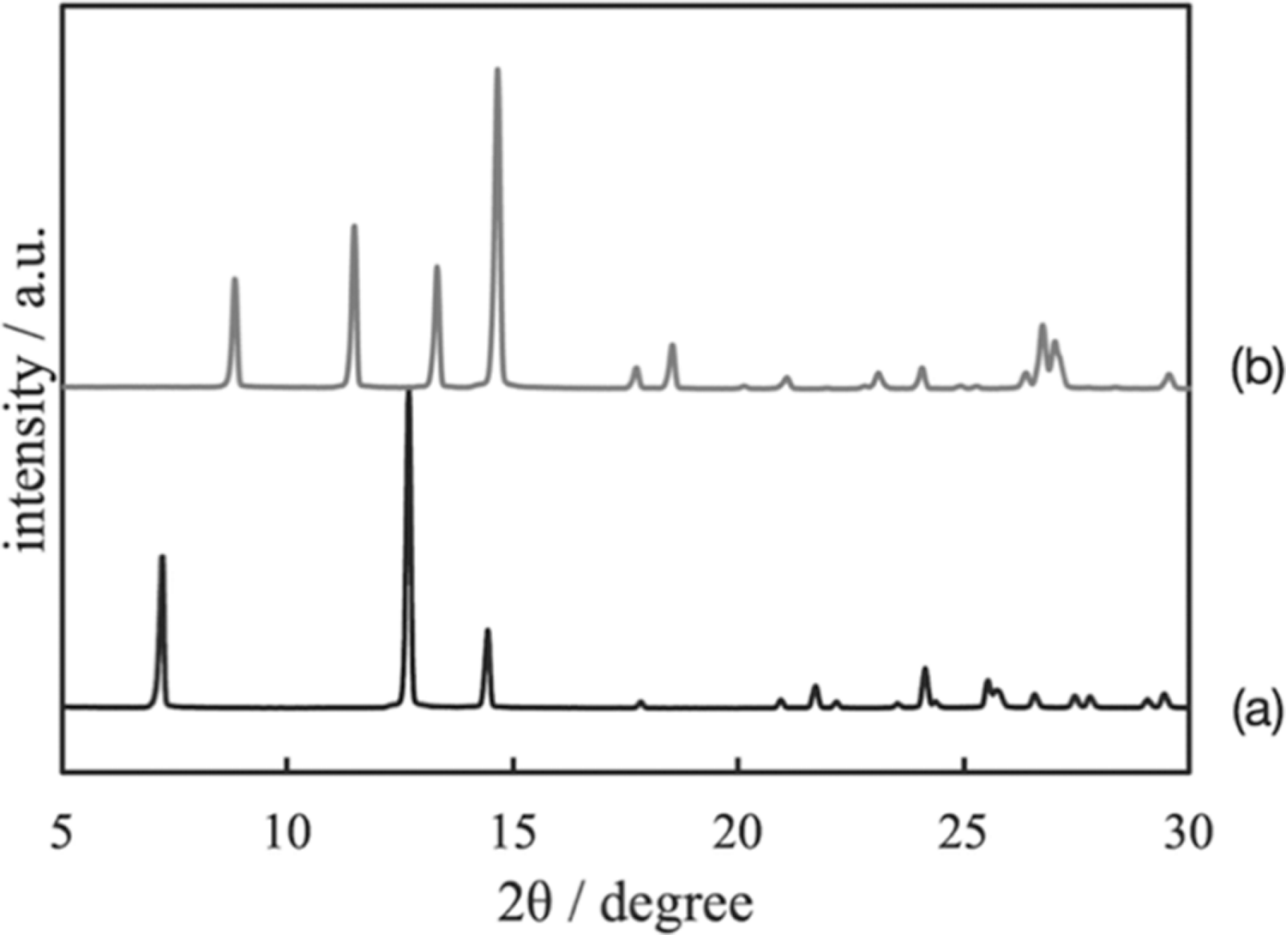
PXRD patterns of (a) theophylline (TPL) and (b) TPL left in water for 5 min.

Figure 2 shows the ATR-FTIR images of the distribution of TPL during tablet dissolution. The distribution of TPL was mainly visualized as red and pink colors in the image at 0 min, while it was mainly visualized as yellow and green colors in the image at 5 min. The area of a solid TPL phase gradually decreased in the ATR-FTIR images from 5 to 90 min. The number of pixels whose normalized integrated absorbance was shown from 0 to 0.1 also gradually increased as shown in Figure S1. These results mean that TPL dissolved in water gradually. Figure 3 shows the time dependence of the IR spectra that were an average of all raw spectra extracted from area *A* shown in Figure 2. Area *A* was a square with a side length of 10 pixels. The bottom-right corner of area *A* was positioned at the point 10 pixels diagonally above from bottom-right corner of the entire image. The peak attributed to TPL, guided by the gray bar, shifted gradually toward a longer wavelength. The wavenumber at the peak maximum was 1708 cm^–1^ at the dry measurement, whereas it was 1699 cm^–1^ at 5 min. As shown in Figure S2, these similar peak shifts were also observed in the IR spectra extracted from area *B* shown in Figure 2. From the previous research, it has been already studied that TPL anhydrate transforms into TPL monohydrate in water.^27^ Previous research reported that a peak located around 1736 and 1688 cm^–1^ shifted from 1713 to 1692 cm^–1^ due to the transformation of the TPL crystal structure.^33^ Additionally, TPL, which was left in water for 5 min, showed new peaks in the PXRD measurement as shown in Figure 4. These new peaks were assigned to TPL monohydrate because their angle positions agreed with TPL monohydrate.^28,29^ As shown in Figure 3, it is suggested that the peak at 1708 cm^–1^ at the dry measurement is assigned to TPL anhydrate and that the peak at 1699 cm^–1^ at 5 min is assigned to TPL monohydrate. The measurements using this ATR-FTIR spectroscopic imaging method can achieve the dissolution behavior of API observed by ATR-FTIR images and the transformation of API during dissolution, clarified by the IR spectra extracted within images.

### Dissolution Test for the Molecular Crystal Composed of Multiple Components

**Figure 5:**
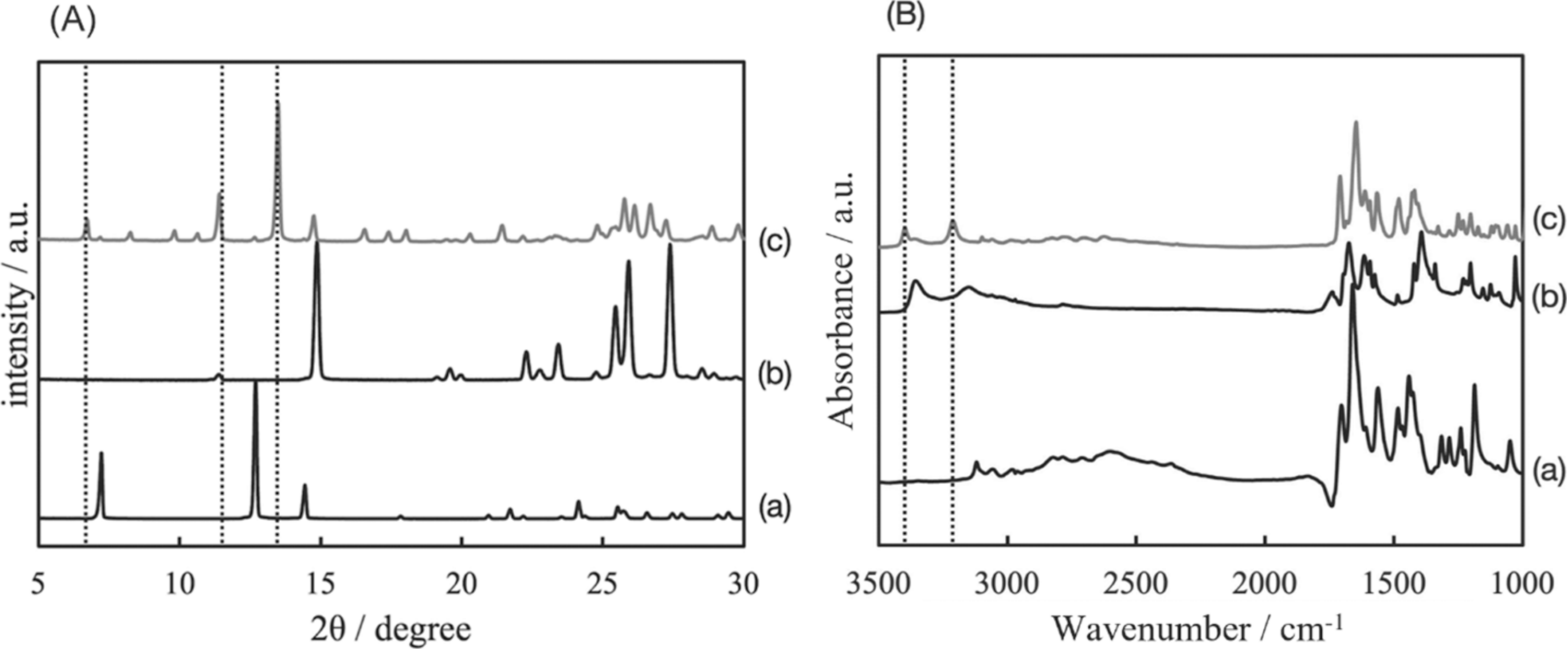
(A) PXRD patterns of (a) theophylline (TPL), (b) nicotinamide (NA), and (c) the sample fabricated by the stirring of TPL and NA. Characteristic peaks of the cocrystal are guided by dotted lines. (B) FTIR spectra of (a) TPL, (b) NA, and (c) the sample fabricated by the stirring of TPL and NA. The intermolecular bonds are guided by dotted lines.

**Figure 6:**
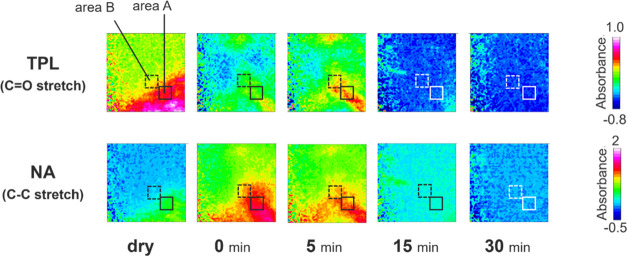
ATR-FTIR spectroscopic images for the distribution of components in a cocrystal during dissolution. The cocrystal is composed of theophylline (TPL) and nicotinamide (NA).

**Figure 7:**
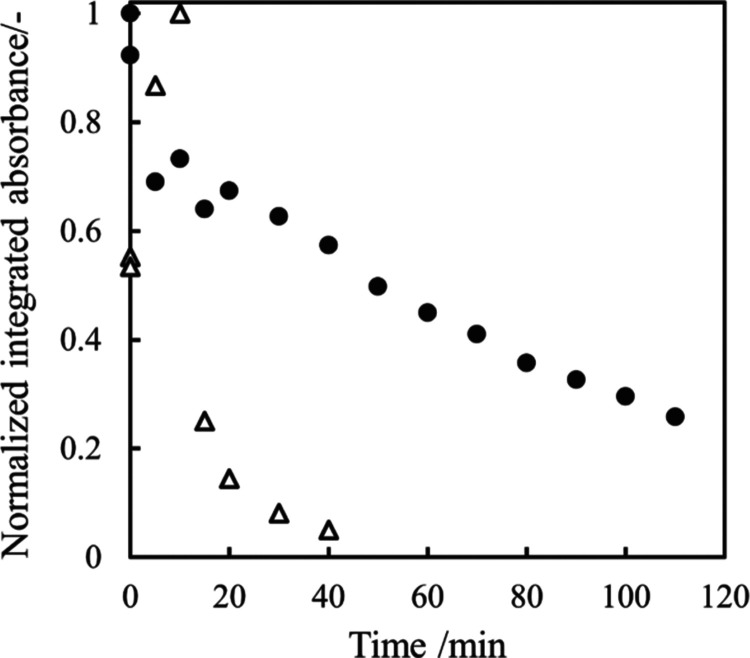
Time dependence for normalized integrated absorbance of the characteristic peak attributed to theophylline (TPL) from the ATR-FTIR images. Both measurements at dry and 0 min are plotted at time = 0. The black circle means TPL (Figure 2) only, and the white triangle means TPL included in the cocrystal (Figure 6).

The cocrystal consisting of TPL as the API and NA as the coformer was selected as a model molecular crystal consisting of multiple components. This system was applied to study the dissolution of the API using ATR-FTIR spectroscopic imaging. The characterization of a sample fabricated by the stirring of TPL and NA was confirmed by PXRD and FTIR measurements before starting the dissolution test of the cocrystal. Figure 5 shows the PXRD patterns and FTIR spectra of TPL, NA, and the powder sample that was fabricated by the stirring of TPL and NA. As shown in Figure 5A, the sample showed new peaks that were assigned to the cocrystal composed of TPL and NA because their angle positions agreed with the cocrystal composed of TPL and NA.^35,36^ As shown in Figure 5B, the sample showed new peaks, guided by the dotted lines, around 3200 and 3400 cm^–1^. These new peaks were assigned to the intermolecular bond between the API and the coformer.^36^ These results suggest that a cocrystal is successfully formed. Figure 6 shows the ATR-FTIR images for the distribution of TPL and NA during the tablet dissolution of cocrystals composed of TPL and NA. Both TPL and NA almost disappeared from the measurement area of ATR-FTIR images in 30 min. For most pixels of ATR-FTIR images in 30 min, the normalized integrated absorbance assigned to TPL and NA showed values between 0 and 0.2, as shown in Figure S3. These results show that TPL and NA almost dissolve in water simultaneously although it is reported that pure NA^37^ has a much higher solubility in water than pure TPL.^38^ Moreover, Figure 7 shows the time dependence of normalized integrated absorbance for the API-only system (Figure 2) and API in the cocrystal system (Figure 6). The rate of API removal in the cocrystal system was faster than that in the API-only system. It means that the dissolution rate of API in the cocrystal system is faster than that in the API system. Hence, the cocrystallization can achieve the enhancement for the dissolution of TPL into water.

**Figure 8:**
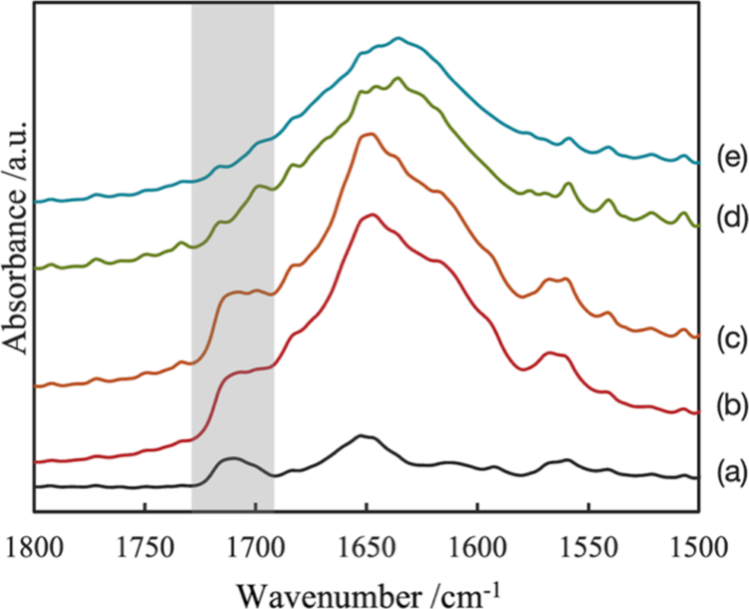
Time dependence of IR spectra. IR spectra at each time condition were averages of all spectra extracted within area *A* in Figure 6. The peak around 1736 and 1688 cm^–1^ was guided by the gray bar: (a) dry, (b) 0 min, (c) 5 min, (d) 15 min, and (e) 30 min.

The color in area *A* in Figure 6 was green in the image of the TPL distribution at 0 min, while it was orange mostly in the image of the TPL distribution at 5 min. To investigate the color shifts in area *A*, the time dependence of IR spectra was analyzed as shown in Figure 8. Area *A* was a square with a side length of 10 pixels. The bottom-right corner of area *A* was positioned at the point 10 pixels diagonally above from the bottom-right corner of the entire image. The IR spectra at each time condition were an average of all spectra extracted from area *A* in each ATR-FTIR image. The peak attributed to TPL, guided by the gray bar, shifted gradually toward a longer wavelength. The wavenumber at the peak maximum was 1711 cm^–1^ for the dry measurement. It was assigned to the cocrystal or the TPL anhydrate because the wavenumber almost agreed with the cocrystal (1712 cm^–1^)^36^ and the TPL anhydrate (1713 cm^–1^).^33^ On the other hand, the wavenumber at the peak maximum was 1699 cm^–1^ at 5 min later. It was assigned to the TPL monohydrate because the wavenumber almost agreed with the TPL monohydrate.^33^ These results of peak shifts imply that the precipitation of TPL is confirmed. Also, area *B* in Figure 6 had a similar peak shift to area *A*, as shown in Figure S4. Therefore, during the dissolution of the cocrystal, the precipitation of API would be partially confirmed as its stable crystal structure in water.

From the results of both ATR-FTIR spectroscopic images (Figure 6) and the IR spectra (Figure 8), the dissolution mechanism of cocrystals can be explained by the following three steps. First, coformer (NA) molecules may dissolve in water, which triggers the breakdown of the crystal structure of the cocrystal. Second, API (TPL) molecules seem to be in a metastable state.^39,40^ Then, API with the metastable state shows a higher solubility in water than its original crystal structure.^39^ Simultaneously, API could be transformed into a stable structure. Therefore, the measurement data with ATR-FTIR spectroscopic imaging can not only help observe the dissolution behavior of the molecular crystal composed of multiple components by ATR-FTIR images but also help understand the transformation of API included in the cocrystal by the IR spectra extracted from images.

## Conclusions

In this study, we applied ATR-FTIR spectroscopic imaging to the tablet dissolution of pharmaceutical molecular crystals: the molecular crystal of a single component and that comprising multiple components. In the dissolution test, we used TPL for the molecular crystal of a single component. The IR spectra extracted from ATR-FTIR images indicated that the crystal structure of TPL transformed from anhydrate to monohydrate in water. Moreover, we used a cocrystal comprising TPL and NA in the dissolution test for a molecular crystal composed of multiple components. The time dependence of ATR-FTIR images showed that TPL and NA were dissolved in water simultaneously. During cocrystal dissolution, the peak of TPL monohydrate can be detected from the IR spectra extracted from the ATR-FTIR images. These results indicate that cocrystallization has the potential to improve the solubility of poorly soluble APIs. However, minor amounts of API precipitation were observed during the dissolution of cocrystals. Overall, ATR-FTIR spectroscopic imaging can provide a visualization of drug dissolution from the tablet matrix and characterize the transformation of molecular crystals. This is expected to provide insights into the dissolution mechanism of pharmaceutical molecular crystals.

## Supporting Information Available

Results of integrated absorbance and time dependence of spectra on theophylline dissolution as well as of integrated absorbance and time dependence of spectra on cocrystal dissolution (PDF)
