# Supplementary material for: Analysis of the Dissolution Behavior of Theophylline and Its Cocrystal Using ATR-FTIR Spectroscopic Imaging
Source: Mol Pharm. 2024 May 28;21(7):3233–9. doi: 10.1021/acs.molpharmaceut.4c00002 (PMC11220746; doi:10.1021/acs.molpharmaceut.4c00002)
Supplement: Supplementary file 1 — mp4c00002_si_001.pdf [file mp4c00002_si_001.pdf]

Supporting information for

Analysis for dissolution behavior of theophylline and its cocrystal using ATR-FTIR spectroscopic imaging

Yuna Tatsumi[a], Yusuke Shimoyama[a*], Sergei G. Kazarian[b*]

[a] Department of Chemical Science and Engineering, Tokyo Institute of Technology, S1-33 2-12-1 Ookayama, Meguro-ku, Tokyo, 1528550, Japan

[b] Department of Chemical Engineering, Imperial College London, London, SW7 2AZ, U.K.

Caption

Figure S1 Histogram results for theophylline dissolution of normalized integrated absorbance between 1736 and 1688 cm^{-1}. (a) dry, (b) 0 min, (c) 5 min, (d) 15 min, (e) 30 min, (f) 50 min and (g) 90 min.

Figure S2 Time dependence of spectra. Spectra at each time condition were average of all spectra extracted within the area B in Figure 2. The area B was a square with a side length of 10 pixels. The bottom-right corner of area B was positioned at the point 20 pixels diagonally above from the bottom-right corner of the entire image. The peak around 1736 and 1688 cm^{-1} was guided by the grey bar. (a) dry, (b) 0 min, (c) 5 min, (d) 15 min, (e) 30 min, (f) 50 min and (g) 90 min.

Figure S3 (A) Histogram results for cocrystal during dissolution of normalized integrated absorbance between 1736 and 1688 cm^{-1}. (B) Histogram results for cocrystal during dissolution of normalized integrated

absorbance between 1447 and 1370 cm^{-1}. (a) dry, (b) 0 min, (c) 5 min, (d) 15 min and (e) 30 min.

Figure S4 Time dependence of spectra. Spectra at each time condition were average of all spectra extracted within the area B in Figure 6. The area B was a square with a side length of 10 pixels. The bottom-right corner of area B was positioned at the point 20 pixels diagonally above from the bottom-right corner of the entire image. The peak around 1736 and 1688 cm^{-1} was guided by the grey bar. (a) dry, (b) 0 min, (c) 5 min, (d) 15 min and (e) 30 min.

Figure

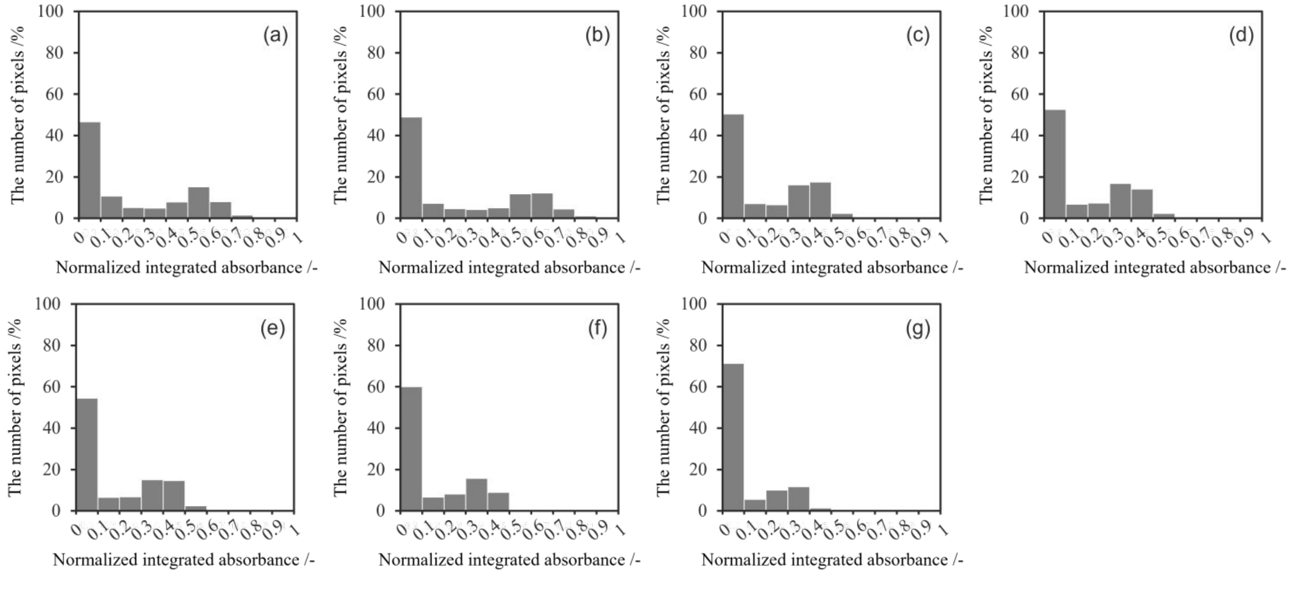

Figure S1 Histogram results for theophylline dissolution of normalized integrated absorbance between 1736 and 1688 cm^{-1}. (a) dry, (b) 0 min, (c) 5 min, (d) 15 min, (e) 30 min, (f) 50 min and (g) 90 min.

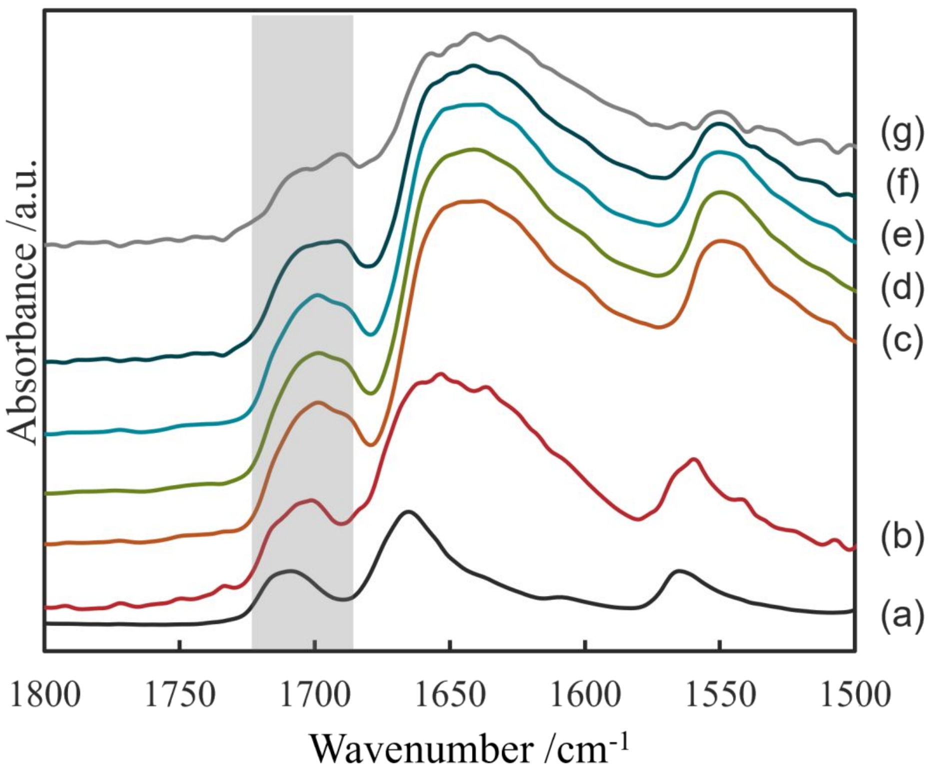

Figure S2 Time dependence of spectra. Spectra at each time condition were average of all spectra extracted within the area B in Figure 2. The area B was a square with a side length of 10 pixels. The bottom-right corner of area B was positioned at the point 20 pixels diagonally above from the bottom-right corner of the entire image. The peak around 1736 and 1688 cm^{-1} was guided by the grey bar. (a) dry, (b) 0 min, (c) 5 min, (d) 15 min, (e) 30 min, (f) 50 min and (g) 90 min.

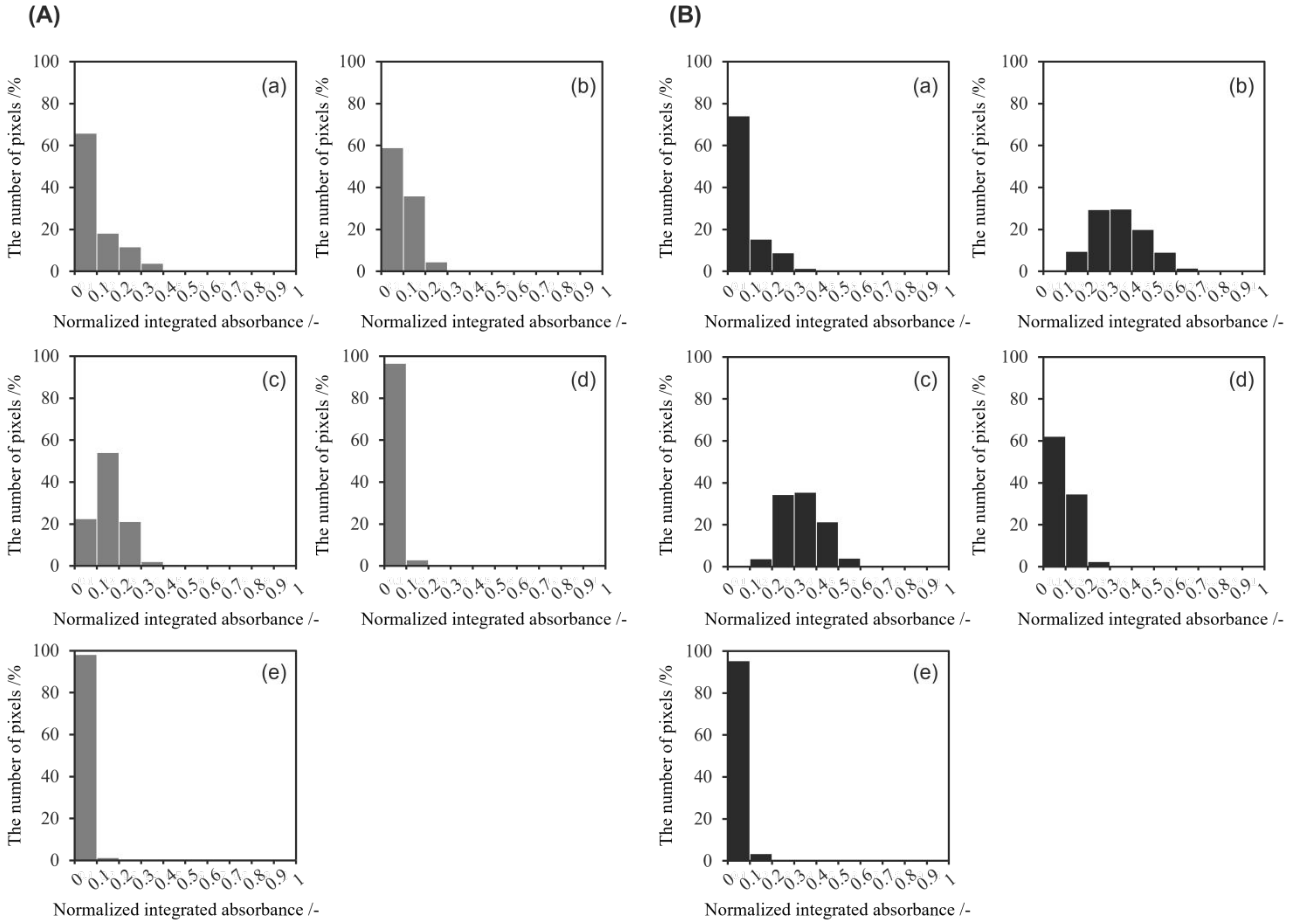

Figure S3 (A) Histogram results for cocrystal during dissolution of normalized integrated absorbance between 1736 and 1688 cm^{-1}. (B) Histogram results for cocrystal during dissolution of normalized integrated absorbance between 1447 and 1370 cm^{-1}. (a) dry, (b) 0 min, (c) 5 min, (d) 15 min and (e) 30 min.

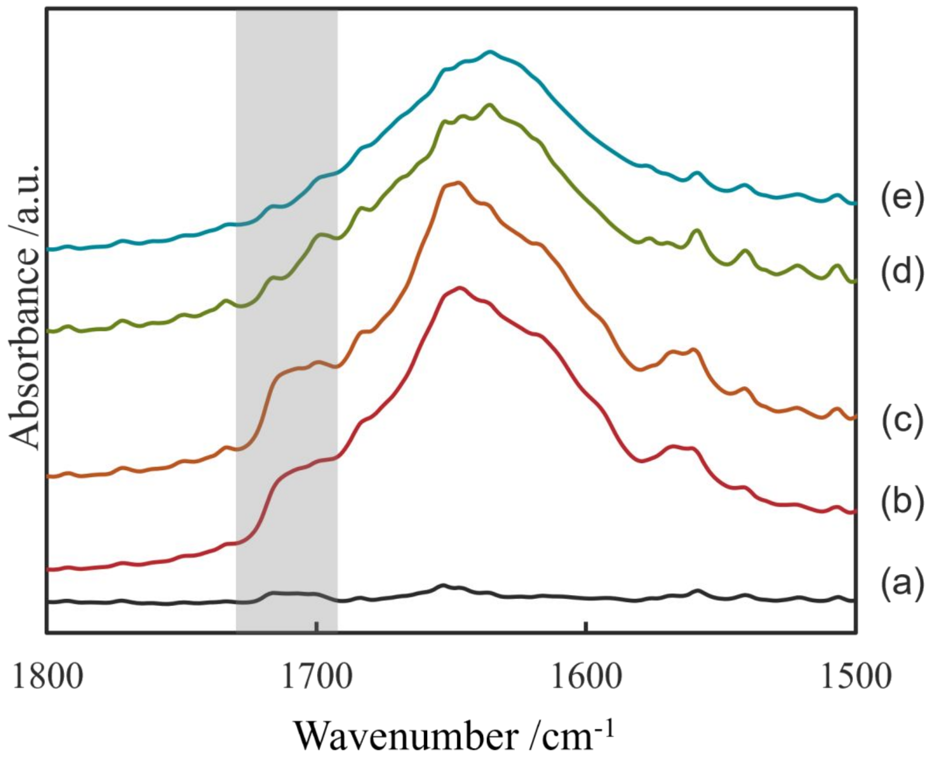

Figure S4 Time dependence of spectra. Spectra at each time condition were average of all spectra extracted within the area B in Figure 6. The area B was a square with a side length of 10 pixels. The bottom-right corner of area B was positioned at the point 20 pixels diagonally above from the bottom-right corner of the entire image. The peak around 1736 and 1688 cm^{-1} was guided by the grey bar. (a) dry, (b) 0 min, (c) 5 min, (d) 15 min and (e) 30 min.
